# Associations between Fine and Coarse Particles and Mortality in Mediterranean Cities: Results from the MED-PARTICLES Project

**DOI:** 10.1289/ehp.1206124

**Published:** 2013-05-17

**Authors:** Evangelia Samoli, Massimo Stafoggia, Sophia Rodopoulou, Bart Ostro, Christophe Declercq, Ester Alessandrini, Julio Díaz, Angeliki Karanasiou, Apostolos G. Kelessis, Alain Le Tertre, Paolo Pandolfi, Giorgia Randi, Cecilia Scarinzi, Stefano Zauli-Sajani, Klea Katsouyanni, Francesco Forastiere, the MED-PARTICLES Study group.

**Affiliations:** 1 Department of Hygiene, Epidemiology and Medical Statistics, Medical School, University of Athens, Athens, Greece; 2 Department of Epidemiology Lazio Region, Rome, Italy; 3 Air Pollution Epidemiology Section, Office of Environmental Health Hazard Assessment, California Environmental Protection Agency, Oakland, California, USA; 4 Centre for Research in Environmental Epidemiology, Barcelona Biomedical Research Park, Barcelona, Spain; 5 Environmental Health Department, French Institute for Public Health Surveillance, Saint-Maurice, France; 6 National School of Public Health, Carlos III Health Institute, Madrid, Spain; 7 Institute of Environmental Assessment and Water Research, Barcelona, Spain; 8 Environmental Department, Municipality of Thessaloniki, Thessaloniki, Greece; 9 Epidemiology Observatory, Department of Public Health, Local Health Authority, Bologna, Italy; 10 Epidemiology Unit, Local Health Authority, Milan, Italy; 11 Department of Epidemiology and Environmental Health, Regional Environmental Protection Agency, Piedmont, Italy; 12 Regional Centre for Environment and Health, Regional Agency for Environmental Prevention of Emilia-Romagna, Modena, Italy

**Keywords:** coarse particles, fine particles, Mediterranean, mortality, particulate matter, time series

## Abstract

Background: Few studies have investigated the independent health effects of different size fractions of particulate matter (PM) in multiple locations, especially in Europe.

Objectives: We estimated the short-term effects of PM with aerodynamic diameter ≤ 10 μm (PM_10_), ≤ 2.5 μm (PM_2.5_), and between 2.5 and 10 μm (PM_2.5–10_) on all-cause, cardiovascular, and respiratory mortality in 10 European Mediterranean metropolitan areas within the MED-PARTICLES project.

Methods: We analyzed data from each city using Poisson regression models, and combined city-specific estimates to derive overall effect estimates. We evaluated the sensitivity of our estimates to co-pollutant exposures and city-specific model choice, and investigated effect modification by age, sex, and season. We applied distributed lag and threshold models to investigate temporal patterns of associations.

Results: A 10-μg/m^3^ increase in PM_2.5_ was associated with a 0.55% (95% CI: 0.27, 0.84%) increase in all-cause mortality (0–1 day cumulative lag), and a 1.91% increase (95% CI: 0.71, 3.12%) in respiratory mortality (0–5 day lag). In general, associations were stronger for cardiovascular and respiratory mortality than all-cause mortality, during warm versus cold months, and among those ≥ 75 versus < 75 years of age. Associations with PM_2.5–10_ were positive but not statistically significant in most analyses, whereas associations with PM_10_ seemed to be driven by PM_2.5_.

Conclusions: We found evidence of adverse effects of PM_2.5_ on mortality outcomes in the European Mediterranean region. Associations with PM_2.5–10_ were positive but smaller in magnitude. Associations were stronger for respiratory mortality when cumulative exposures were lagged over 0–5 days, and were modified by season and age.

## Introduction

A large number of epidemiological studies have reported evidence of adverse health effects of airborne particulate matter (Katsouyanni et al. 2009; Pope and Dockery 2006), leading the scientific community to investigate in more detail the role of specific characteristics of particles. Recent studies have attributed previously reported associations with particulate matter with aerodynamic diameter ≤ 10 μm (PM_10_) mainly to effects of smaller particles—those with diameter ≤ 2.5 μm (PM_2.5_) (Klemm et al. 2000; Laden et al. 2000; Ostro et al. 2006; Zanobetti and Schwartz 2009; Zhou et al. 2011). Potential effects of coarse particles with diameter between 2.5 and 10 μm (PM_2.5–10_) are still under investigation, although evidence of adverse effects is also accumulating (Brunekreef and Forsberg 2005; Meister et al. 2012; Perez et al. 2009). The great majority of previous studies have been conducted in the United States, including many multicity studies, whereas the few European studies that have been undertaken have been based on single cities (Atkinson et al. 2010; Jiménez et al. 2009; Ostro et al. 2011; Perez et al. 2009). Location-specific characteristics, such as the higher prevalence of diesel vehicles, particularly passenger cars, and the higher density of the population in European cities compared with North American ones, may result in different exposure patterns. Furthermore, most of the U.S. studies have been based not on daily PM measures, but on PM measures every 3 or 6 days, which could lead to misclassification and possibly an underestimation of the short-term effects of particles on health outcomes (Katsouyanni et al. 2009; Klemm et al. 2011). Data specific to Europe are also needed to inform the revision in air quality standards for Europe that has been announced.

In the framework of the LIFE+ MED-PARTICLES project, which aims to characterize particulate pollution and its health effects across the European Mediterranean countries, we have undertaken the first European multicity investigation of the short-term effects of PM_2.5_ and PM_2.5–10_ exposure on mortality outcomes. Here we report the results of our analysis of acute exposure to PM in 10 metropolitan areas in this region [see Supplemental Material, Figure S1 (http://dx.doi.org/10.1289/ehp.1206124)], including estimated effects by cause of death, and according to age group, sex, and season.

## Data and Methods

*Data*. Data are from 12 cities (Marseille, France; Athens and Thessaloniki, Greece; Bologna, Milan, Modena, Parma, Reggio Emilia, Rome, and Turin, Italy; and Barcelona and Madrid, Spain). These cities represent 10 metropolitan areas in the European Mediterranean region, including the Italian Emilia Romagna (ER) region that comprises Modena, Reggio Emilia, and Parma, three small cities with similar air pollution levels, climate, and population density.

For each city, data were collected on daily counts of all-cause mortality {excluding deaths from external causes [*International Classification of Diseases, 9th* and *10th Revisions* (ICD-9, ICD-10)] (WHO 1999): ICD-9 codes 001–799, ICD-10 codes A00–R99} for all ages and according to sex and age < 75 or ≥ 75 years, in addition to daily counts of cardiovascular mortality (ICD-9 codes 390–459, ICD10 codes I00–I99) and respiratory mortality (ICD-9 codes 460–519, ICD10 codes J00–J99). Data from each city covered at least 3 consecutive years during 2001–2010.

Daily PM_2.5_, PM_10_, and gaseous pollutant data [sulfur dioxide (SO_2_, 24 hr), nitrogen dioxide (NO_2_, 24 hr), and ozone (O_3_, 8 hr)] were provided by monitoring networks in each participating city. All measurements were made using the gravimetric method or an equivalent method (beta-attenuation), except for measurements made using the tapered element oscillating microbalance (TEOM) method in Marseille. Because the focus of the analysis was on fine (PM_2.5_) and coarse (PM_2.5–10_) particles, and because PM_2.5–10_ was estimated as the difference between PM_10_ and PM_2.5_, the study period was selected based on the availability and completeness of PM_2.5_ concentration data. These data were obtained from urban or suburban background sites or, when appropriate as a measure of the exposure of nearby population, from fixed monitors located near traffic. All measurement sites included in the study were required to have at least 75% complete information for the study period. To obtain city-specific exposure estimates, the monitor-specific concentrations were averaged, and missing values from the averaged series were imputed as the average of the values of the remaining stations for that day multiplied by a factor equal to the ratio of the annual mean for the missing station over the corresponding annual mean from the other stations (Katsouyanni et al. 2001). When data were missing from all relevant monitoring stations on a given day, measurements were classified as missing for that day. PM_10_ measurements for all areas except Bologna and Turin were from the same fixed monitoring stations that provided PM_2.5_ measurements, to maintain comparability and allow the calculation of monitor-specific time-series data for PM_2.5–10_. On average, PM_2.5_ and PM_2.5–10_ data were missing on 4% of days during the study (ranging from 0% in Thessaloniki to 12% in Marseille), and PM_10_ data were missing for 2% of study days (ranging from 0% in Thessaloniki to 5% in Bologna). Missing data were at random, as confirmed by local authorities and by data inspection because there were no patterns observed.

Time-series data on daily temperature (degrees Celsius, daily mean) were used to control for the potential confounding effects of weather. External information on influenza epidemics was also collected, if available from hospital admissions records.

*Methods*. We used a hierarchical modeling approach. First, we fit separate regression models for each city to allow location-specific control for seasonal effects, weather, and other potential confounders. We then used the results of the individual city analysis in a meta analysis to derive overall estimates.

PM-mortality associations for each city were estimated using Poisson regression models allowing for overdispersion. The city-specific model is of the form:

log *E*[*Y_t_^c^*] = β_0_*^c^* + *b^c^* × *PM_t_^c^* + *s^c^*(time*_t_^c^*,df) + *__i__*Σs*_i_^c^*(*x_it_^c^*,df*_i_*) + γX, [1]

where *E*[*Y_t_^c^*] is the expected value of the Poisson distributed variable *Y_t_^c^* indicating the daily mortality count on day *t* at city *c* with variance (*Y_t_^c^*) = ϕ⊇*E* [*Y_t_^c^*], ϕ represents the overdispersion parameter, *x_it_^c^* is the value of the *x_i_* meteorological covariate on day *t* in city *c*, and *PM_t_^c^* is the particulate matter metric concentration on day *t* in city *c*. The smooth functions *s* capture the nonlinear relationship between the time-varying covariates, calendar time, and daily mortality using corresponding degrees of freedom (df). We used penalized regression splines as implemented by Wood (2000) in R, with natural cubic splines as basis functions. We chose *k* = 50 basis functions and eight effective degrees of freedom (edfs) per year of available data to control for seasonality and obtain conservative estimates of effect (Katsouyanni et al. 2009; Samoli et al. 2008). To control for weather, the time-series models included smooth terms for temperature on the day of death and the day before death in using a natural spline with 3 df (Katsouyanni et al. 2009). We also included dummy variables for the day of the week, holidays, and influenza epidemics, represented by the vector X in Equation 1. In cities with no influenza data available, we included a dummy variable that was assigned the value of 1 when the 7-day moving average of respiratory mortality was greater than the 90th percentile of the city-specific distribution. Because this method of controlling for influenza was based on the distribution of respiratory mortality, we adjusted for influenza dummy variable only when modeling all-cause and cardiovascular mortality in these cities (Samoli et al. 2006). Finally, we controlled for the decrease in populations during the summer vacation period (typical of Mediterranean cities) using a three-level ordinal variable assigned a value of 2 during the 2-week period around mid-August, 1 from July 16 to August 31 (with the exception of the aforementioned 2-week period), and 0 (the reference category) on the remaining days (Stafoggia et al. 2010).

The pollutant was entered in the model using one of three cumulative lags chosen *a priori* to represent immediate effects (lag 0–1), delayed effects (lag 2–5), and weekly effects (lag 0–5). We also fitted a cubic polynomial distributed lag model over a period of 8 days to investigate the shape of each association (Zanobetti et al. 2002). Further analyses used the cumulative lag that produced the strongest effect estimates for each outcome based on the meta-analytic polynomial distributed lag shape and on the pooled estimates for each lag structure. This strategy was considered an appropriate compromise between *a priori* definitions and flexibility of lag choice for different exposure/outcome combinations (Stafoggia et al. 2010).

Further analyses of associations with each PM exposure were implemented using the chosen reference lag for each outcome. To evaluate how sensitive our results were to the choice of the degree of smoothing for seasonality control we also applied Poisson models using two alternative methods: *a*) using penalized splines with the degrees of smoothing for seasonality selected to minimize the absolute value of the sum of the partial autocorrelations of the residuals from lags one to 30 [PACF (partial autocorrelation function) criterion] with a minimum of 3 df per year (Katsouyanni et al. 2009), and *b*) using a case-crossover approach by modeling the time trend in the Poisson models with a three-way interaction between year, month, and day of death (Lu et al. 2008) that accounted for all possible combinations of year, month, and day of week, without additional adjustment for the day of the week. This is equivalent to the standard case-crossover design with a time-stratified strategy used to select control days on the same day of the week within the same month and year of the event day.

To investigate potential confounding by other pollutants, we used two-pollutant models of associations with PM_2.5_ or PM_2.5–10_ that were adjusted for NO_2_ (24 hr), SO_2_ (24 hr), O_3_ (8 hr), or PM_2.5_ or PM_2.5–10_. We also investigated modification of associations between all-cause mortality and PM by age group (< 75 or ≥ 75 years of age) and sex in separate analyses.

We explored seasonal variation in associations by fitting separate Poisson regression models for cooler months (October–March) and warmer months (April–September) using 4 df/year to adjust for seasonality within each period.

Finally, we carried out threshold analyses to investigate exposure–response relationships between PM_2.5_ or PM_2.5–10_ and all-cause mortality (Samoli et al. 2008). We selected a grid of threshold values in increments of 5 μg/m^3^ from 0 to 35 μg/m^3^ for PM_2.5_, and from 0 to 20 μg/m^3^ for PM_2.5–10_ (i.e., 0, 5, 10 …μg/m^3^). For each threshold value *h*, we fit a threshold model to the data for the available cities that included the term *x_t_* + = *x_t_* if *x_t_* > 0 and 0 otherwise, where *x_t_* = PM*_t_* – *h*, PM is the PM concentration on day *t*, and *h* is the threshold value. We then computed the deviance of the fitted model for all cities for a given threshold value, and the average deviance for that threshold over all cities. We repeated the analysis for all threshold values to identify a possible threshold that minimized the mean deviance.

In the second stage of the analysis, we assumed that city-specific effect estimates were normally distributed around an overall estimate. We derived pooled random-effects estimates with the random variance component estimated by iteratively reweighted least squares (Berkey et al. 1995). We used chi-square tests and the *I*^2^ statistic (Higgins and Thompson 2002) to examine heterogeneity. There was a general agreement between the two measures concerning the amount of observed heterogeneity attributed to the between-cities variability.

All models were fit in R version 15.0 (R Development Core Team, Vienna, Austria). Results are presented as the estimated percent change in the outcome associated with a 10-μg/m^3^ increase in PM, or an increase in PM equal to the median of the distribution of the city-specific interquartile ranges (IQR) for the exposure being assessed.

Statistical significance was considered at α⊇< 0.05.

## Results

Table 1 presents descriptive characteristics by metropolitan area. Together, these areas comprise a population of > 14 million people. The mean daily total number of deaths ranged from 11 (in Bologna) to 81 (in Athens). For respiratory mortality, daily deaths ranged from 1 (in Bologna and Emilia Romagna) to 10 (in Madrid). All 10 areas provided data for PM_2.5_ with median levels ranging from 13.6 μg/m^3^ (in Madrid) to 27.7 μg/m^3^ (in Thessaloniki), whereas for eight areas with available collocated measurements, median PM_10_ and PM_2.5–10_ concentrations ranged from 25.0 μg/m^3^ and 8.0 μg/m^3^, respectively, in Marseille, compared with 44.4 μg/m^3^ and 15.8 μg/m^3^ in Thessaloniki. There was relatively small variability among cities in the levels of gaseous pollutants and in mean daily temperature.

**Table 1:** Descriptive characteristics of the cities in MED-PARTICLES.

City	Study period	Population × 1,000	Mean no. of deaths per day			Median (25th–75th percentile)					Mean temperature (°C)
			All-cause	CVD	Respiratory	PM_2.5_ (μg/m^3^)	PM_2.5–10_ (μg/m^3^)	PM_10_ (μg/m^3^)	NO_2_ (μg/m^3^)	O_3_ (μg/m^3^)	
Athens	2007–2009	3,000	81	37	9	21.5 (16.5–27.5)	12.0 (8.0–18.5)	35.0 (26.5–45.5)	45.2 (35.6–56.2)	69.9 (47.1–90.7)	19.0
Barcelona	2003–2009	1,595	38	12	4	22.1 (17.0–29.8)	11.2 (6.6–18.0)	35.4 (26.2–47.0)	38.8 (27.5–50.5)	62.0 (38.1–79.8)	14.7
Bologna	2006–2010	372	11	4	1	20.0 (14.0–32.0)	—	32.0 (24.0–48.0)	49.6 (37.2–62.2)	59.9 (28.8–92.6)	14.7
Emilia Romagna	2008–2010	530	13	5	1	16.7 (11.7–27.0)	11.3 (8.0–15.7)	29.0 (21.0–34.3)	41.9 (30.6–54.1)	68.6 (27.1–104.9)	14.7
Madrid	2007–2009	3,133	60	18	10	13.6 (9.5–18.8)	14.2 (9.7–20.3)	28.5 (19.9–39.4)	53.0 (39.4–69.7)	46.1 (26.5–60.9)	15.2
Marseille	2001–2008	797	22	7	2	16.0 (11.0–22.0)	8.0 (6.0–12.0)	25.0 (19.0–33.0)	48.8 (37.7–60.5)	80.6 (56.0–103.4)	15.6
Milan	2006–2010	1,300	35	12	3	22.7 (14.0–45.7)	13.1 (7.4–19.3)	35.6 (23.5–60.1)	57.8 (43.5–72.9)	55.0 (18.7–92.2)	13.9
Rome	2006–2010	2,719	58	24	4	17.5 (12.9–24.0)	12.5 (8.5–16.7)	30.5 (23.1–39.5)	59.5 (46.1–70.8)	72.3 (40.4–95.5)	15.9
Thessaloniki	2007–2009	613	18	8	2	27.7 (22.0–34.7)	15.8 (11.3–18.1)	44.4 (35.5–56.5)	43.6 (32.2–56.5)	56.0 (38.9–79.2)	15.9
Turin	2006–2010	908	21	8	2	24.0 (14.0–48.0)	—	37.5 (24.8–65.8)	56.1 (41.3–74.0)	66.6 (23.9–104.6)	12.7
CVD, cardiovascular diseases.											

The correlation between PM_2.5_ and PM_2.5–10_ ranged from 0.2 (in Barcelona and Marseille) to 0.7 (in Thessaloniki), and the correlation between PM_2.5_ and NO_2_ ranged from 0.3 (in Barcelona) to 0.8 (in Milan) [see Supplemental Material, Table S1 (http://dx.doi.org/10.1289/ehp.1206124)]. Correlations between PM_2.5_ or PM_2.5–10_ and SO_2_ and O_3_ were < 0.4 in all cities except Milan and Turin, where the correlations were around 0.6 in absolute value.

**Figure 1:**
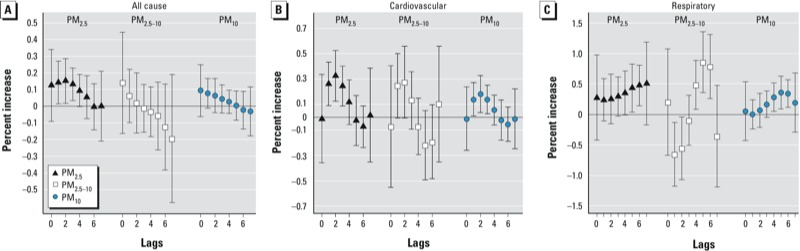
Results from second-stage random-effects models from city-specific polynomial distributed lag models (adjusted for seasonality, temperature, day of the week, holidays, influenza, and summer population decrease) for lags 0–7 presented as percent increase (95% CI) in all-cause (*A*), cardiovascular (*B*), and respiratory mortality (*C*) per 10-μg/m^3^ increase in PM.

Figure 1 presents estimates from the distributed lag models for all-cause, cardiovascular, and respiratory mortality. In general, the strongest positive associations with respiratory and cardiovascular mortality were delayed compared with associations estimated for all-cause mortality, although associations with respiratory and cardiovascular mortality were highly variable depending on the lag period, particularly for PM_2.5–10_ and respiratory mortality.

Table 2 presents the estimated percent change in all-cause, cardiovascular, and respiratory mortality associated with a 10-μg/m^3^ increase in PM for cumulative lags of 0–1, 2–5, and 0–5 days. PM_2.5_ had statistically significant positive associations with all mortality outcomes for all lags except for respiratory mortality at lag 0–1, which was positive but not statistically significant. Associations between PM_2.5_ and respiratory and cardiovascular mortality were stronger than associations with all-cause mortality. In general, associations with PM_2.5_ increased for longer cumulative lag periods; for example, a 10-μg/m^3^ increase in PM_2.5_ on the day of death and the previous day was associated with a 0.55% increase (95% CI: 0.27, 0.84%) in all-cause mortality, whereas a 10-μg/m^3^ increase in PM_2.5_ 0–5 days before death was associated with a 0.70% increase (95% CI: 0.22, 1.18%).

**Table 2:** Percent increase (95% CI) in mortality outcomes associated with 10-μg/m^3^ increase in PM for different cumulative lag structures.^*a*^

Association under investigation	Lag 0–1	Lag 2–5	Lag 0–5
All-cause mortality
PM_2.5_	0.55 (0.27, 0.84)	0.51 (0.07, 0.96)	0.70 (0.22, 1.18)
PM_2.5–10_	0.30 (–0.10, 0.69)	–0.03 (–0.70, 0.64)*	0.07 (–0.75, 0.90)*
PM_10_	0.32 (0.13, 0.52)	0.19 (–0.18, 0.56)*	0.28 (–0.14, 0.71)*
Cardiovascular mortality
PM_2.5_	0.57 (0.07, 1.08)	0.77 (0.20, 1.34)	0.86 (0.15, 1.57)
PM_2.5–10_	0.28 (–0.37, 0.93)	0.33 (–0.59, 1.26)	0.33 (–0.78, 1.46)
PM_10_	0.31 (–0.01, 0.62)	0.41 (0.04, 0.79)	0.54 (0.09, 0.99)
Respiratory mortality
PM_2.5_	0.72 (–0.11, 1.55)	1.63 (0.62, 2.65)	1.91 (0.71, 3.12)
PM_2.5–10_	–0.13 (–1.25, 1.01)	0.72 (–0.31, 1.76)	0.76 (–0.70, 2.25)
PM_10_	0.23 (–0.35, 0.81)	1.14 (0.28, 2.00)	1.12 (0.29, 1.95)
^***a***^Results from second-stage random-effects models pooling estimates from city-specific Poisson models adjusted for seasonality, temperature, day of the week, holidays, influenza, and summer population decrease. *Statistically significant heterogeneity as indicated by *p* < 0.10 from Cochran’s Q and *I*^2 ^> 50%.			

Associations were positive but not statistically significant for PM_2.5–10_ and all three mortality outcomes, except for associations between respiratory mortality and lag 0–1 exposure, and between all-cause mortality and lag 2–5 exposure, which were essentially null (Table 2). Whereas an association between PM_2.5–10_ and all-cause mortality was limited to immediate exposure (lag 0–1: 0.30%; 95% CI: –0.10, 0.69%), associations with cardiovascular mortality were similar for all three cumulative lag periods (e.g., lag 0–5: 0.33%; 95% CI: –0.78, 1.46%), and associations with respiratory mortality were limited to the longer cumulative exposure periods (e.g., lag 0–5: 0.76%; 95% CI: –0.70, 2.25%).

PM_10_ was positively associated with all mortality outcomes, with a statistically significant association for all cause mortality for lag 0–1 (Table 2). For respiratory and cardiovascular mortality, associations were stronger and statistically significant for longer periods of cumulative exposure.

There was no statistically significant heterogeneity observed in the effects of particles on mortality outcomes between the Mediterranean cities [see Supplemental Material, Table S2 (http://dx.doi.org/10.1289/ehp.1206124)], except for the association between PM_10_ and total mortality after longer periods of exposure.

Based on these findings, for all PM exposures, further analyses of all-cause mortality focused exposure on the same day and the previous day (lag 0–1), whereas cumulative exposure through the previous 5 days (lag 0–5) was used for respiratory and cardiovascular mortality. Increases in lag 0–1 exposure equal to the median of the city-specific interquartile ranges for PM_2.5_ (13 μg/m^3^) and PM_2.5–10_ (11 μg/m^3^) were associated with estimated increases in all-cause mortality of 0.72% (95% CI: 0.35, 1.09%) and 0.33% (95% CI: –0.10, 0.76%), respectively. The same cumulative increases in PM_2.5_ and PM_2.5–10_ over the same day and previous 5 days (lag 0–5) were associated with 1.11% (95% CI: 0.19, 2.04%) and 0.37% (95% CI: –0.86, 1.61%) increases in cardiovascular mortality, and 2.49% (95% CI: 0.92, 4.07%) and 0.84% (95% CI: –0.77, 2.48%) increases in respiratory mortality. When analyses of PM_2.5_ were restricted to the eight areas that also provided PM_10_ data (i.e., excluding Turin and Bologna) estimated increases associated with a 13-μg/m^3^ increase in exposure were 0.76% (95% CI: 0.31, 1.20%) for all-cause mortality (lag 0–1) and 1.50% (95% CI: 0.42, 2.58%) and 3.82% (95% CI: 1.77, 5.91%) for cardiovascular and respiratory mortality, respectively (lag 0–5).

**Figure 2:**
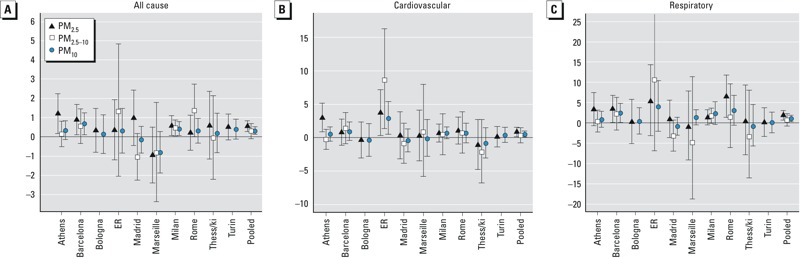
Percent increase (95% CI) in mortality outcomes associated with 10-μg/m^3^ increases in PM for each metropolitan area and overall. Results from models using 8 df/year for seasonality control for all-cause (lag 0–1) (*A*), cardiovascular (lag 0–5) (*B*), and respiratory (lag 0–5) (*C*) mortality. Abbreviations: ER, Emilia Romagna; Thess/ki, Thessaloniki. City-specific estimates are also adjusted for temperature, day of the week, holidays, influenza, and summer population decrease.

Figure 2 presents city-specific and pooled effects estimated as the percent change in mortality outcomes associated with a 10-μg/m^3^ increase in PM_2.5_, PM_2.5–10_, and PM_10_, respectively. Although the single city estimates are less precise and are often not statistically significant, associations with PM_2.5_ exposures were generally higher in larger metropolitan areas [see also Supplemental Material, Table S3 (http://dx.doi.org/10.1289/ehp.1206124)].

Estimates from two-pollutant models (Table 3) provided evidence of confounding for associations between PM_2.5–10_ and mortality. Associations with fine particles were less affected by adjustment for other pollutants, although there was some indication of confounding by NO_2_, and SO_2_. When the three cities with correlations > 0.70 between PM_2.5_ and NO_2_ were excluded [Bologna, Milan, and Turin; see Supplemental Material, Table S1 (http://dx.doi.org/10.1289/ehp.1206124)], the NO_2_-adjusted association between PM_2.5_ and all-cause mortality increased (0.40% increase; 95% CI: –0.25, 1.06%, compared with 0.28%; 95% CI: –0.12, 0.68% when all cities were included, lag 0–1). Associations between PM_2.5_ and mortality increased with adjustment for PM_2.5–10_ (Table 3). As for PM_2.5_ and PM_2.5–10_, associations between PM_10_ and mortality (data not shown) also decreased when adjusted for NO_2_ or SO_2_ (0.13%; 95% CI: –0.11, 0.37% and 0.10%; 95% CI: –0.22%, 0.43%, for total mortality respectively, compared with 0.32%; 95% CI: 0.13, 0.52% without adjustment).

**Table 3:** Percent increase (95% CI) in mortality associated with 10‑μg/m^3^ increase in fine and coarse particles for selected lag periods.^*a*^

Primary pollutant	Second pollutant	All-cause mortality (lag 0–1)	Cardiovascular mortality (lag 0–5)	Respiratory mortality (lag 0–5)
PM_2.5_
	None	0.55 (0.27, 0.84)	0.86 (0.15, 1.57)	1.91 (0.71, 3.12)
	+SO_2_	0.33 (–0.37, 1.03)	0.56 (–0.60, 1.74)	1.98 (–0.01, 4.01)
	+NO_2_	0.28 (–0.12,0.68)	0.64 (–0.30, 1.60)	2.15 (0.40, 3.94)
	+O_3_	0.46 (0.16, 0.76)	0.94 (0.16, 1.73)	2.45 (0.94, 3.97)
	+PM_2.5–10_	0.59 (0.00, 1.18)*	1.35 (0.44, 2.26)	3.44 (1.63, 5.29)
PM_2.5–10_
	None	0.30 (–0.10, 0.69)	0.33 (–0.78, 1.46)	0.76 (–0.70, 2.25)
	+SO_2_	0.13 (–0.40, 0.66)	–0.09 (–1.30, 1.13)	–1.01 (–4.30, 2.38)
	+NO_2_	0.06 (–0.53, 0.66)	–0.17 (–1.27, 0.95)	–0.25 (–2.91, 2.45)
	+O_3_	0.22 (–0.50, 0.95)	0.21 (–1.11, 1.55)	–0.19 (–2.60, 2.29)
	+PM_2.5_	–0.05 (–0.84, 0.75)	–0.28 (–1.36, 0.81)	–0.85 (–2.81, 1.15)
^***a***^Results from second-stage random-effects models pooling estimates from city-specific 2-pollutant Poisson models adjusted for seasonality, temperature, day of the week, holidays, influenza, and summer population decrease. *Statistically significant heterogeneity as indicated by *p* < 0.10 from Cochran’s Q and *I*^2^> 50%.				

Estimates from our primary analyses, which used penalized regression splines with 8 df/year to adjust for seasonal variation, were conservative relative to estimates from models that controlled for seasonality using the PACF criterion or a case-crossover analysis (Table 4), except for the estimated association between PM_10_ and respiratory mortality under the case-crossover model. In addition, associations between PM_2.5–10_ and all-cause mortality were statistically significant based on both alternative models, and were increased from the primary model estimates to a greater extent than the corresponding estimates for PM_2.5_. All associations were substantially stronger for exposures during warm months (April–September) compared with colder months (October–March) (Table 4).

**Table 4:** Percent increase (95% CI) in mortality associated with 10-μg/m^3^ increases in PM: sensitivity analysis results from single-pollutant models.^*a*^

Association under investigation	Statistical methods for seasonality control in city-specific models^*b*^			Seasonal analysis	
	8 df/year	PACF	Case-crossover	Warm period (4 df/year)	Cold period (4 df/year)
All-cause mortality (lag 0–1)
PM_2.5_	0.55 (0.27, 0.84)	0.97 (0.64, 1.30)	0.71 (0.37, 1.04)	2.24 (1.46, 3.03)	0.23 (–0.08, 0.54)
PM_2.5–10_	0.30 (–0.10, 0.69)	0.89 (0.31, 1.47)*	0.61 (0.10, 1.13)	0.57 (–0.16,1.31)	0.26 (–0.43, 0.95)
PM_10_	0.31 (0.10, 0.52)	0.64 (0.37, 0.90)	0.46 (0.22, 0.70)	1.09 (0.51, 1.67)	0.17 (–0.09, 0.43)
Cardiovascular mortality (lag 0–5)
PM_2.5_	0.86 (0.15, 1.57)	1.84 (1.06, 2.63)	0.99 (0.24, 1.75)	2.60 (0.73, 4.51)	0.48 (–0.26, 1.22)
PM_2.5–10_	0.33 (–0.78, 1.46)	1.78 (0.46, 3.12)	0.53 (–0.52, 1.60)	0.48 (–1.21, 2.20)	–0.20 (–1.40, 1.02)
PM_10_	0.57 (0.08, 1.06)	1.25 (0.75, 1.76)	0.62 (0.15, 1.10)	1.18 (0.10, 2.27)	0.26 (–0.23, 0.75)
Respiratory mortality (lag 0–5)
PM_2.5_	1.91 (0.71, 3.12)	3.21 (1.67, 4.78)	2.84 (1.36, 4.33)	6.46 (2.60, 10.47)	1.74 (0.27, 3.24)
PM_2.5–10_	0.76 (–0.70, 2.25)	1.63 (–0.90, 4.23)	0.91 (–0.99, 2.86)	1.21 (–2.02, 4.55)	0.30 (–1.82, 2.46)
PM_10_	1.24 (0.37, 2.12)	1.81 (0.72, 2.92)	1.27 (0.37, 2.18)	2.48 (0.38, 4.62)	0.80 (–0.15, 1.76)
^***a***^Results from second-stage random-effects models pooling estimates from city-specific single pollutant Poisson models adjusted for seasonality, temperature, day of the week, holidays, influenza, and summer population decrease. ^***b***^City-specific models adjusted for seasonality using penalized splines with 8 df/year, dfs estimated by the PACF criterion, or a three-way interaction to fit the case-crossover design. *Statistically significant heterogeneity as indicated by *p* < 0.10 from Cochran’s Q and *I*^2 ^> 50%.					

Table 5 presents associations with all-cause mortality according to age group (< 75 or ≥ 75 years) and sex. Associations between a 10-μg/m^3^ increase in PM_2.5_ and mortality appeared to be limited to the older age group (0.77%; 95% CI: 0.43, 1.10% compared with 0.02%; 95% CI: –0.51, 0.55% for those < 75, *p* = 0.02). In contrast, the opposite pattern was observed for PM_2.5–10_ (0.10%; 95% CI: –0.47, 0.68% among those ≥ 75 years vs. 0.76%; 95% CI: 0.03, 1.49% for those < 75 years, *p* = 0.16). All associations were slightly stronger in males than in females, but differences were not statistically significant (*p* = 0.59–0.83).

**Table 5:** Percent increase (95% CI) in mortality associated with 10-μg/m^3^ increase in particles: effect modification by age and sex of the associations between particles (lag 0–1) and all-cause mortality.^*a*^

All-cause mortality	PM_2.5_	*p*-Value for interaction	PM_2.5–10_	*p*-Value for interaction	PM_10_	*p*-Value for interaction
All ages, both sexes	0.55 (0.27, 0.84)		0.30 (–0.10, 0.69)		0.31 (0.10, 0.52)
By age (years)		0.02		0.16		0.24
< 75	0.02 (–0.51, 0.55)		0.76 (0.03, 1.49)		0.14 (–0.21, 0.50)
≥ 75	0.77 (0.43, 1.10)		0.10 (–0.47, 0.68)		0.40 (0.17, 0.63)
By sex		0.80		0.59		0.83
Male	0.51 (0.09, 0.94)		0.69 (–0.19, 1.59)		0.38 (0.09, 0.67)
Female	0.44 (0.10, 0.79)		0.40 (–0.20, 1.00)		0.34 (0.06, 0.61)
^***a***^Results from second-stage random-effects models pooling estimates from city-specific subgroup analysis by the level of the corresponding effect modifier using single-pollutant Poisson models adjusted for seasonality, temperature, day of the week, holidays, influenza, and summer population decrease.						

Threshold models for all-cause mortality in association with a 10-μg/m^3^ increase in PM_2.5_ or PM_2.5–10_ (lag 0–1) did not support the presence of a threshold because the best-fitting models (with the lowest deviance values) were those that did not assume a threshold [see Supplemental Material, Table S4 (http://dx.doi.org/10.1289/ehp.1206124)].

## Discussion

We investigated associations between mortality outcomes and fine and coarse particles in 10 European Mediterranean metropolitan areas participating in the MED-PARTICLES project. This is the first European multicity analysis to report on these associations using complete daily time-series data on PM_2.5_ and PM_2.5–10_.

PM_2.5_ was statistically significantly associated with all-cause, cardiovascular, and respiratory mortality. Associations estimated for our study populations are slightly weaker but generally consistent with those previously reported for single-city studies in Europe and studies from the U.S. (Atkinson et al. 2010; Klemm et al. 2000; Laden et al. 2000; Ostro et al. 2006; Zanobetti and Schwartz 2009). Specifically, for a 10-μg/m^3^ increase in PM_2.5_ on the same day and the previous day (lag 0–1) we estimated a 0.55% increase in all-cause mortality and a 0.57% increase in cardiovascular mortality. For respiratory mortality, we estimated an increase of 1.91% in association with cumulative exposures over the same day and 5 previous days, suggesting delayed effects. Our estimates are comparable with estimates reported by Ostro et al. (2006) for the same exposure period and increment increase (0.60% increase in all-cause mortality) in nine California counties (with climates similar to the metropolitan areas included in the present study), but are slightly lower than estimates for 112 U.S. cities reported by Zanobetti and Schwartz (2009) (0.98% increase in all-cause mortality). However, Zanobetti and Schwartz (2009) estimated a comparable increase (0.50%) in all-cause mortality for a subset of U.S. cities characterized by a Mediterranean climate, which suggests that variation among studies may be attributable partly to differences in climate.

Compared with short-term associations between fine particles and mortality reported for individual European cities, our pooled estimates of associations with a 10-μg/m^3^ increase in PM_2.5_ were weaker than those reported for Barcelona [for all-cause mortality, Perez et al. (2008) reported a 3.20% increase, whereas Ostro et al. (2011) reported a 1.40% increase] and Madrid [2.80% increase in respiratory mortality for a single-day exposure (Guaita et al. 2011) and a similar increase in deaths from circulatory causes (Maté et al. 2010)]. However, Atkinson et al. (2010) reported no evidence of associations between PM_2.5_ and all-cause or cardiovascular mortality in London. The higher effects on respiratory mortality with prolonged exposure to fine particles are compatible with findings of previous reports, especially for cause-specific mortality, which, however, have considered PM_10_ and not PM_2.5_ (Pope 2007; Zanobetti et al. 2003). Associations between PM_2.5_ and mortality were stronger for exposures during warmer months, in accordance with previous U.S. findings (Zanobetti and Schwartz 2009), possibly attributed to differential time–activity patterns, with Mediterranean population spending more time outdoors, and better exposure characterization of the population. The overall association between PM_2.5_ and all-cause mortality in our study population appears to be driven by an effect among people ≥ 75 years of age, consistent with findings from California (Ostro et al. 2006) suggesting that the elderly are the most susceptible population subgroup. Finally, there was no statistically significant heterogeneity observed in our results due to the similar characteristics between the areas.

Associations between short-term PM_2.5_ exposures (lag 0–1) and all-cause mortality appeared to be confounded by NO_2_ and SO_2_, although positive associations persisted when adjusted for other individual pollutants. Since both PM_2.5_ and NO_2_ are primarily traffic-derived pollutants (Querol et al. 2012) that are highly correlated, it is difficult to estimate their independent effects. In contrast with all-cause mortality, associations between PM_2.5_ (lag 0–5) and respiratory mortality increased when adjusted for other pollutants, which may reflect delayed effects of PM_2.5_ compared with effects of other pollutants on respiratory deaths. The associations between PM_2.5_ and all three outcomes were stronger when adjusted for PM_2.5–10_, but estimates from these models were unstable because of the high correlation between the two pollutants. Zanobetti and Schwartz (2009) reported no evidence of confounding between fine and coarse particles.

PM_2.5–10_ exposures were positively, but not significantly, associated with all mortality outcomes (for example, a 10-μg/m^3^ increase at lag 0–1 was associated with 0.30% increase in all-cause mortality). Stronger associations were estimated for some metropolitan areas (e.g., Rome) possibly reflecting less measurement error and better characterization of population exposure in these areas. Zanobetti and Schwartz (2009) reported statistically significant associations of a similar magnitude between PM_2.5–10_ and total or cardiovascular mortality in 47 U.S. cities, but did not find an association in the analysis of the subset of cities characterized by a Mediterranean climate. However, in a multicity study in California, Malig and Ostro (2009) estimated statistically significant associations of PM_2.5–10_ with all-cause and cardiovascular mortality. Regarding previously reported European results, there was no indication of an association in the United Kingdom (Atkinson et al. 2010; Brunekreef and Forsberg 2005), but Perez et al. (2008) reported nonsignificant associations in Barcelona; Meister et al. (2012) reported a stronger and statistically significant association with all-cause mortality in Stockholm (1.68%); and Halonen et al. (2009) reported positive associations with respiratory mortality. When we applied alternative methods for seasonality control in the present analysis, we estimated slightly higher and statistically significant associations with all-cause and cardiovascular mortality. Nevertheless, after adjusting for PM_2.5_ in the alternative models, the associations decreased and became nonsignificant (0.41% increase; 95% CI: –0.51, 1.35% for all cause mortality using the PACF, and 0.18% increase; 95% CI: –0.65, 1.01% using the case-crossover approach), thus supporting the possibility that health effects of PM are driven mainly by effects of fine particles. As was the case with estimates of effect for fine particles, we found stronger associations between PM_2.5–10_ and mortality during warmer months, consistent with findings reported for the U.S. (Zanobetti and Schwartz 2009) but not Stockholm (Meister et al. 2012). Differences may be attributable partly to different sources of coarse particles, such as desert dust transported from the Sahara region to Mediterranean areas, which is more evident in spring and early autumn (Pey et al. 2013). An unexpected finding was the stronger association between PM_2.5–10_ and mortality among those < 75 versus ≥ 75 years of age in the Mediterranean study populations, in contrast with stronger associations with PM_2.5_ in the elderly. One possible explanation might be differences in time–activity patterns according to age and season, combined with greater penetration of fine particles into indoor environments compared with coarse particles. Hence, if the effect of both particle fractions is encountered mainly in the warm period (which is representative of the Mediterranean climate), younger people who spent more time outdoors may be more affected by coarse particles, and older people, who spend comparably more time indoors, may be more affected by fine ones. Still the absence of effect of fine particles among the younger age group requires further investigation.

The nonsignificant positive associations between PM_2.5–10_ and mortality became essentially null when we controlled for other pollutants, which indicate that health effects of PM are a consequence of traffic-related pollution, because the coarse fraction of PM is dominated by dust. Alternatively, because within-city spatial variation is greater for PM_2.5–10_ (due to traffic non-tailpipe emissions) than for PM_2.5_ (Canepari et al. 2008), there is more exposure measurement error for PM_2.5–10_, possibly biasing their effects.

Associations between PM_10_ and mortality in the Mediterranean populations included in our analysis were consistent with findings of previous reports and the effect modification patterns by season, age, and sex (Katsouyanni et al 2001, 2009). As expected, effect estimates for PM_10_ were weaker than those for PM_2.5_ and stronger than those for PM_2.5–10_, but seemed to be driven primarily by fine particles: For an IQR increase in PM_10_, PM_2.5_, and PM_2.5–10_ in the eight cities with data on all three PM metrics, estimated increases in all-cause mortality (lag 0–1) were 0.64%, 0.76%, and 0.33%, respectively, and 2.38%, 3.82%, and 0.84% for respiratory mortality (lag 0–5).

There is considerable toxicological evidence of adverse health effects of PM, including evidence of cytotoxicity and inflammatory effects through increased oxidative stress resulting from exposure to traffic-related particles such as PM_2.5_ (Gualtieri et al. 2010; Park et al. 2011). However, there is also increasing evidence that coarse particles may activate inflammatory pathways (Graff et al. 2009; Karlsson et al. 2011), including an *in vitro* study that reported that coarse particles had an inflammatory potential similar to fine particles on an equal mass basis (Schwarze et al. 2007).

Exposure measurement error is an inherent disadvantage of time-series studies, because the average of selected fixed monitoring stations does not reflect the true average exposure of the population. Nevertheless, there is some evidence that exposure measurement error in time-series analysis tends to bias estimates downward (Zeger et al. 2000). In the MED-PARTICLES project, exposure error was imposed by the limited number of monitors per city and variability due to differences in PM_2.5_ measurement methods among the cities. Moreover, because PM_2.5–10_ was not directly monitored but estimated as the difference between PM_10_ and PM_2.5_, part of its variability may be attributable to measurement error in both PM_10_ and PM_2.5_. On the other hand, the combination of large and full time-series of exposure data on particles coming from multiple locations similar in topography is a major advantage of our study.

## Conclusions

We report evidence of adverse health effects of fine particles on mortality outcomes in the European Mediterranean region. Coarse particle exposures were also positively associated with mortality, but in most models estimates did not reach the nominal level of statistical significance. Overall our findings suggest that exposure to smaller particles, which mostly originate from traffic, has stronger impacts on health than exposure to larger particles. Associations with both PM_2.5_ and PM_2.5–10_ were stronger during warmer months, but associations with fine particles appeared to be limited to those ≥ 75 years of age, whereas associations with coarse particles were stronger among those < 75 years.

## Supplemental Material

(430 KB) PDF
