# Supplementary material for: Associations between Fine and Coarse Particles and Mortality in Mediterranean Cities: Results from the MED-PARTICLES Project
Source: Environ Health Perspect. 2013 May 17;121(8):932–8. doi: 10.1289/ehp.1206124 (PMC3734494; doi:10.1289/ehp.1206124)
Supplement: (430 KB) PDF [file ehp.1206124.s001.pdf]

Environmental Health Perspectives doi:10.1289/ehp.1206124

SUPPLEMENTAL MATERIAL

Associations between Fine and Coarse Particles and Mortality in Mediterranean Cities: Results from the MED-PARTICLES Project

Evangelia Samoli[1], Massimo Stafoggia[2], Sophia Rodopoulou[1], Bart Ostro[3,4], Christophe Declercq[5], Ester Alessandrini[2], Julio Díaz[6], Angeliki Karanasiou[4,7], Apostolos G. Kelessis[8], Alain Le Tertre[5], Paolo Pandolfi[9], Giorgia Randi[10], Cecilia Scarinzi[11], Stefano Zauli-Sajani[12], Klea Katsouyanni[1], and Francesco Forastiere[2]; the MED-PARTICLES Study group.

[1]Department of Hygiene, Epidemiology and Medical Statistics, Medical School, University of Athens, Athens, Greece.

[2]Department of Epidemiology Lazio Region, Rome, Italy

[3]Air Pollution Epidemiology Section, Office of Environmental Health Hazard Assessment, CAL EPA, Oakland, California U.S.A.

[4]Centre for Research in Environmental Epidemiology (CREAL), Barcelona Biomedical Research Park, Barcelona, Spain

[5]Environmental Health Department, French Institute for Public Health Surveillance (InVS), Saint-Maurice, France

[6]National School of Public Health, Carlos III Health Institute, Madrid, Spain

[7]Institute of Environmental Assessment and Water Research (IDAEA-CSIC), Barcelona, Spain

[8]Environmental Department, Municipality of Thessaloniki, Thessaloniki, Greece.

[9]Epidemiology Observatory, Department of Public Health, Local Health Authority, Bologna, Italy

[10]Epidemiology Unit, Local Health Authority, Milan, Italy

[11]Department of Epidemiology and Environmental Health, Regional Environmental Protection Agency, Piedmont, Italy

[12]Regional Centre for Environment and Health, Regional Agency for Environmental Prevention of Emilia-Romagna, Modena, Italy

MED-PARTICLES Study Group: Italy: E. Alessandrini, P. Angelini, G. Berti, L. Bisanti, E. Cadum, M. Catrambone, M. Chiusolo, M. Davoli, F. de' Donato, M. Demaria, M. Gandini, M. Grosa, A. Faustini, S. Ferrari, F. Forastiere, P. Pandolfi, R. Pelosini, C. Perrino, A. Pietrodangelo, L. Pizzi, V. Poluzzi, G. Priod, G. Randi, A. Ranzi, M. Rowinski, C. Scarinzi, M. Stafoggia, E. Stivanello, S. Zauli-Sajani; Greece: K. Dimakopoulou, K. Elefteriadis, K. Katsouyanni, A.G. Kelessis, T. Maggos, N. Michalopoulos, S. Pateraki, M. Petrakakis, S. Rodopoulou, E. Samoli, V. Sypsa; Spain: D. Agis, J. Alguacil, B. Artiñano, J. Barrera-Gómez, X. Basagaña, J. de la Rosa, J. Diaz, R. Fernandez, B. Jacquemin, A. Karanasiou, C. Linares, B. Ostro, N. Perez, J. Pey, X. Querol, AM Sanchez, J. Sunyer, A. Tobias; France: M. Bidondo, C. Declercq, A. Le Tertre, P. Lozano, S. Medina, L. Pascal, M. Pascal.

Table of Contents

Figure S1. Map of participating metropolitan areas in MED-PARTICLES Project. **Page 4**

Table S1. Correlations between $PM_{2.5}$ and the other pollutants according to metropolitan area. **Page 5**

Table S2. I^2 values and p-values for X^2 tests for heterogeneity in the associations between mortality and particles. **Page 6**

Table S3. City-specific percent increase (and 95% confidence intervals (CIs)) in mortality associated with a 10-µg/m^3 increase in fine and coarse particles, for the selected lag structure per association. **Page 7**

Table S4. Results from threshold models for all-cause mortality. Mean deviance and pooled percent increase (and 95% confidence intervals (CIs)) in mortality for a 10-µg/m^3 increase in the average of lags 0-1 exposures in $PM_{2.5}$ and $PM_{2.5-10}$. **Page 8**

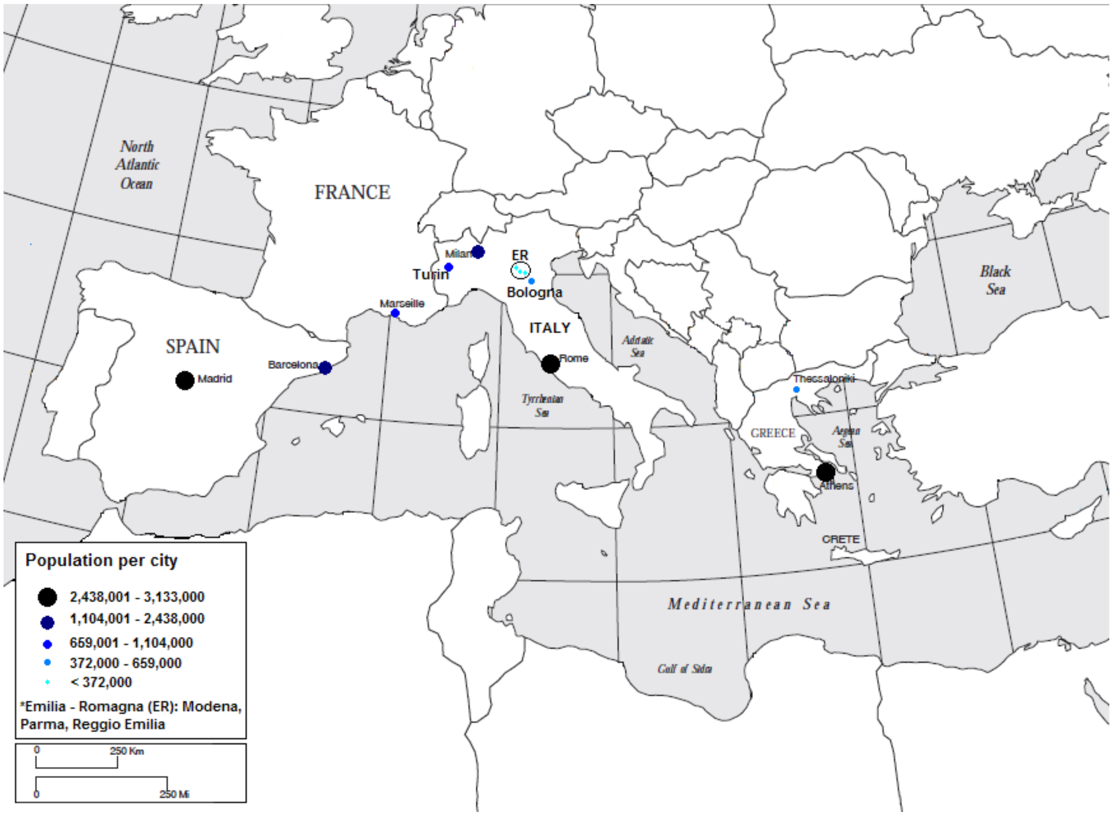

Supplemental Material, Figure S1. Map of participating metropolitan areas in MED-PARTICLES Project.

Supplemental Material, Table S1. Correlations between $PM_{2.5}$ and the other pollutants according to metropolitan area.

City	$PM_{2.5}$-$PM_{2.5\text{-}10}$	$PM_{2.5}$-NO_2	$PM_{2.5}$-SO_2	$PM_{2.5}$-O_3
Athens	0.33	0.41	0.18	0.33
Barcelona	0.19	0.33	0.25	-0.10
Bologna	-	0.71	-	-0.50
ER	0.50	0.69	-	-0.50
Madrid	0.65	0.62	0.17	-0.27
Marseille	0.20	0.69	0.31	-0.03
Milan	0.41	0.76	-	-0.65
Rome	0.33	0.67	0.42	-0.26
Thess/ki	0.68	0.55	-	-0.19
Turin	-	0.72	0.60	-0.63

Supplemental Material, Table S2. I^2 values and p-values for X^2 tests for heterogeneity in the associations between mortality and particles.

Association under investigation	Lag 0-1		Lag 2-5		Lag 0-5	
	I^2	p	I^2	p	I^2	p
**All-cause mortality**						
**$PM_{2.5}$**	0	0.59	29	0.18	17	0.28
**$PM_{2.5\text{-}10}$**	25	0.23	59	0.02	61	0.01
**PM_{10}**	0	0.53	55	0.02	52	0.03
**Cardiovascular mortality**						
**$PM_{2.5}$**	11	0.34	0	0.49	6	0.39
**$PM_{2.5\text{-}10}$**	0	0.95	16	0.30	15	0.31
**PM_{10}**	0	0.54	0	0.69	0	0.58
**Respiratory mortality**						
**$PM_{2.5}$**	0	0.52	0	0.43	0	0.61
**$PM_{2.5\text{-}10}$**	27	0.22	0	0.56	11	0.35
**PM_{10}**	0	0.95	27	0.20	0	0.51

Supplemental Material, Table S3. City-specific precent increase (and 95% confidence intervals (CIs)) in mortality associated with a 10-μg/m^3 increase in fine and coarse particles, for the selected lag structure per association.[a]

City	$PM_{2.5}$ Total mortality lag 0-1	$PM_{2.5}$ Cardiovascular mortality lag 0-5	$PM_{2.5}$ Respiratory mortality lag 0-5	$PM_{2.5-10}$ Total mortality lag 0-1	$PM_{2.5-10}$ Cardiovascular mortality lag 0-5	$PM_{2.5-10}$ Respiratory mortality lag 0-5
Athens	1.20 (0.16, 2.25)	2.97 (0.85, 5.14)	3.32 (-0.66, 7.46)	0.15 (-0.51, 0.82)	-0.26 (-1.73, 1.23)	0.44 (-2.22, 3.17)
Barcelona	0.89 (0.11, 1.68)	0.81 (-1.12, 2.78)	3.42 (0.13, 6.82)	0.55 (-0.35, 1.46)	1.41 (-0.89, 3.77)	2.22 (-1.69, 6.30)
Bologna	0.32 (-0.81, 1.47)	-0.38 (-3.04, 2.35)	0.23 (-5.07, 5.84)	.	.	.
Emilia-Romagna	0.35 (-1.20, 1.94)	3.71 (0.35, 7.18)	5.28 (-3.08, 14.36)	1.33 (-2.05, 4.83)	8.58 (1.39, 16.28)	10.62 (-6.86, 31.39)
Madrid	0.98 (-0.45, 2.43)	0.31 (-3.13, 3.88)	0.92 (-3.59, 5.63)	-1.05 (-2.25, 0.16)	0.86 (-3.81, 2.18)	-3.21 (-6.95, 0.69)
Marseille	-0.95 (-2.39, 0.50)	0.28 (-3.43, 4.14)	-0.97 (-9.12, 7.91)	-0.83 (-3.37, 1.78)	0.86 (-5.79, 7.99)	-4.83 (-18.71, 11.42)
Milan	0.58 (0.06, 1.10)	0.68 (-0.65, 2.04)	2.42 (-0.36, 5.27)	0.98 (-0.13, 2.09)	0.45 (-2.55, 3.54)	2.56 (-3.68, 9.21)
Rome	0.21 (-0.69, 1.13)	1.03 (-0.94, 3.05)	6.49 (1.41, 11.83)	1.36 (-0.01, 2.74)	0.72 (-2.31, 3.84)	1.50 (-6.07, 9.67)
Thessaloniki	0.59 (-1.15, 2.36)	-1.09 (-4.77, 2.72)	0.39 (-7.82, 9.32)	-0.06 (-2.21, 2.14)	-2.12 (-6.74, 2.73)	-3.31 (-13.49, 8.06)
Turin	0.52 (-0.15, 1.19)	0.14 (-1.38, 1.68)	0.16 (-3.27, 3.72)	.	.	.

[a]Results from Poisson models adjusted for seasonality, temperature, day of the week, holidays, influenza and summer population decrease.

Supplemental Material, Table S4. Results from threshold models for all-cause mortality. Mean deviance and pooled percent increase (and 95% confidence intervals (CIs)) in mortality for a 10-µg/m^3 increase in the average of lags 0-1 exposures in $PM_{2.5}$ and $PM_{2.5\text{-}10}$. [a]

	$PM_{2.5}$		$PM_{2.5\text{-}10}$	
Threshold at	Deviance	% (95%CI)	Deviance	% (95%CI)
0 µg/m^3	1563.55	0.53 (0.24, 0.82)	1333.45	0.31 (-0.23, 0.86)
5 µg/m^3	1563.55	0.53 (0.24, 0.82)	1333.49	0.30 (-0.25, 0.85)
10 µg/m^3	1563.57	0.53 (0.24, 0.82)	1333.53	0.28 (-0.29, 0.85)
15 µg/m^3	1563.67	0.53 (0.22, 0.83)	1333.66	0.31 (-0.26, 0.89)
20 µg/m^3	1563.93	0.50 (0.18, 0.82)	133.53	0.34 (-0.032, 1.01)
25 µg/m^3	1564.43	0.44 (0.09, 0.79)	-	-
30 µg/m^3	1564.81	0.37 (-0.01, 0.75)	-	-
35 µg/m^3	1564.85	0.32 (-0.09, 0.74)	-	-

[a]Results from second stage random-effects models pooling estimates from city-specific Poisson models adjusted for seasonality, temperature, day of the week, holidays, influenza and summer population decrease.
